# Effect of Amalgam Restorations and Operation Parameters on Diagnostic Accuracy of Caries Detection Using Cone Beam Computed Tomography: An In Vitro Study

**DOI:** 10.1155/2021/2679012

**Published:** 2021-08-16

**Authors:** Mohamed Mehanny, Rahaf A. AlMohareb, Marcel Noujeim

**Affiliations:** ^1^Basic Dental Science, College of Dentistry, Princess Nourah Bint Abdulrahman University, Riyadh, Saudi Arabia; ^2^Clinical Dental Science Department, College of Dentistry, Princess Nourah Bint Abdulrahman University, Riyadh, Saudi Arabia; ^3^Department of Comprehensive Dentistry, University of Texas Health Science Center, San Antonio, Texas, USA

## Abstract

This study was aimed to evaluate the diagnostic accuracy of cone beam computed tomography (CBCT) in detecting noncavitated approximal caries at different exposure parameters and to assess the impact of artifacts generated by amalgam restorations in an in vitro study. Seventy-eight approximal surfaces of extracted teeth were prepared with intentionally created noncavitated approximal caries of different depths; then, thirteen teeth with class 2 amalgam restorations were replaced with one tooth with normal surfaces in each block. CBCT volumes for all teeth were acquired using a Planmeca Promax 3D Mid imaging unit before and after placement of amalgam teeth, with different exposure parameters at low and high definition, both applying and omitting the Metal Artifact Reduction algorithm. The lesions were classified into four groups with regard to lesion extension. All teeth underwent histological analysis as gold standard. The histological examination showed that the distribution of lesions was as follows: 39.8% sound, enamel lesions of less and more than half the enamel thickness each 17.8%, and 24.6% dentin lesions. The detection sensitivity was found to be 0.972%, and specificity was found to be 0.937% for the detection of noncavitated approximal initial enamel and dentin caries. The highest diagnostic accuracy was found when using operating parameters of 90 kV_p_, 8 mA, and high resolution (75 *μ*m) with nonamalgam teeth; all modes showed statistically significant higher AUCs than mode 2 (80 kV_p_, 7 mA, and 75 *μ*m). However, for teeth with amalgam restorations, the highest accuracy was obtained at low resolution (200 *μ*m) with the other parameters kept the same. It could be concluded that increasing the peak voltage and current improves diagnostic accuracy for the detection of noncavitated approximal caries. Moreover, diagnostic accuracy was found to be higher upon using high spatial resolution when diagnosing caries without adjacent amalgam restorations. There is a statistically significant difference with and without amalgam with respect to all modes.

## 1. Introduction

The early signs of approximal caries can be detected visually as discoloration or coarseness at the site, as well as radiographically. Although discoloration and approximal surface coarseness may indicate caries, detection of carious lesions by direct observation is difficult, if not impossible. Therefore, radiography is very important for the detection of approximal caries, being 88% more efficient than direct observation [1, 2].

Early detection of noncavitated enamel lesions is of high significance, because caries progression can be ceased at this stage, and dental tissue can also be preserved with minimally invasive approaches and without the need for restorative treatment [3, 4]. On the other hand, the challenges of clinical detection of approximal caries prior to cavitation and the ability to distinguish between the presence or absence of a carious lesion (i.e., sensitivity and specificity), become important when imaging methods are used. Working with devices and modalities with high sensitivity and specificity for the detection of approximal caries enables prompt, accurate diagnosis of actual caries and avoids unnecessary cavitation surgery in a noncaries tooth [5].

Recently, the accuracy of cone beam computed tomography (CBCT) for detecting demineralization in approximal surfaces has been explored in in vitro studies [6, 7], which discovered that the depth of lesions imaged with CBCT (3D Accuitomo) corresponded well with that observed in histological sections. While others found no benefit in overall accuracy for CBCT over the intraoral film and digital receptors for detecting demineralization in approximal surfaces [8–10].

As with other technologies, there are recognized limitations to CBCT, including the potential for increased radiation, artifact formation, scatter, noise, and dose variations within a volume of interest that affect image quality [11]. Image quality, as measured by the contrast-to-noise ratio (CNR), can be improved by adjusting various scan parameters such as scan field of view (FOV) [12], electrical current (mA) [13], tube voltage (kV_p_) [14]. To reduce possible degradation of CBCT imaging, Metal Artifact Reduction (MAR) algorithms have been developed [15].

The usefulness of CBCT in cariology, especially in the diagnosis of early noncavitated carious lesions, is still under investigation and its effectiveness is currently controversial. This study is aimed therefore to assess the diagnostic accuracy of CBCT in detecting noncavitated approximal caries at different exposure parameters and to evaluate the influence of artifacts caused by amalgam restorations on the final image quality in vitro.

## 2. Materials and Methods

This study protocol was approved by Princess Nourah Bint Abdulrahman University Institutional Review Board, Riyadh, Saudi Arabia (approval no. 20-0561).

### 2.1. Teeth Selection

**Figure 1:**
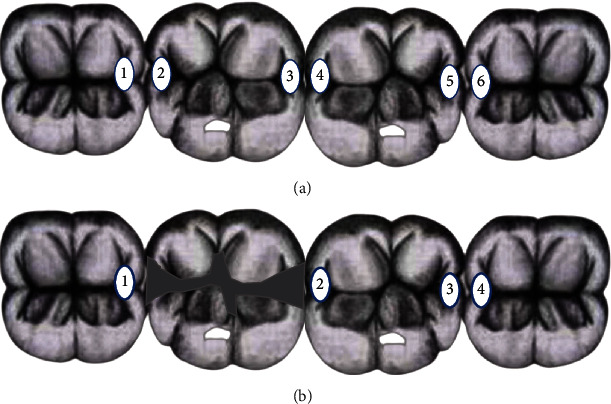
Diagram representing the numbering system of included surfaces within the block. (a) Block with teeth without amalgam restoration. (b) Block after the addition of MOD amalgam restoration to one tooth.

We obtained 52 extracted human sound posterior teeth and divided them into 13 blocks of four teeth each embedded in rubber base blocks in anatomical positions to establish approximal surfaces in contact, resulting in 78 sound approximal surfaces in contact as the noncontact outer proximal surfaces were not counted. We excluded 5 surfaces that had been broken during the caries induction procedure, leaving 73 in-contact surfaces for examination. Each block has 6 approximal surfaces that are in contact with one another. The contact surfaces were numbered from 1 to 6, surface 1 being the first contact surface on the left, and surface 6 being the first contact surface on the right (Figure 1(a)).

**Figure 2:**
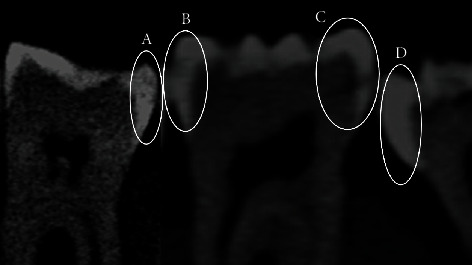
Sagittal CBCT images of the surfaces with different lesion depth and sound surface. (a) D1 Caries depth. (b) D2 Caries depth. (c) D3 Caries depth. (d) Sound surface.

Of the 73 surfaces, 13 were prepared with a D1 carious lesion (less than 1/2 enamel thickness), 13 surfaces with a D2 carious lesion (more than 1/2 enamel thickness but not involving the dentino-enamel junction (DEJ), 17 surfaces with a D3 lesion (involving the DEJ and dentin), and 30 surfaces were left sound (Figure 2). The lesions were created using an acidified gel (0.1 M lactic acid and 0.1 M NaOH mixed to a pH of 4.5, followed by the addition of 6% (*w*/*v*) hydroxyethyl cellulose) [16]. The acidified gel is known to imitate the characteristics of enamel bacterial plaques, creating cyclical demineralization and remineralization and therefore early caries.

All teeth, except predetermined circles on approximal surfaces where lesions were to be created, were protected by a varnish mask, then immersed for 14 days in the gel demineralizing solution to create D1 lesions. The teeth were then removed, cleaned, and revarnished, except for teeth where D2 and D3 lesions were to be created, then returned to the gel for a further 14 days. A further removal, cleaning, revarnishing, and immersion was finally conducted to create D3 lesions. All teeth were returned to their places in the blocks for CBCT acquisition.

Afterwards, 13 teeth with amalgam class II MOD restorations (width of 1/4 the intercuspal distance, depth of 2.5 mm occlusal depth, and 3.5 mm proximal depth) were replaced with teeth with normal surfaces, and one amalgam tooth was added to each block to evaluate its effect on caries detection in the neighboring teeth, examining 4 surfaces and numbering them from 1 to 4 as shown in Figure 1(b).

### 2.2. CBCT Examination

**Figure 3:**
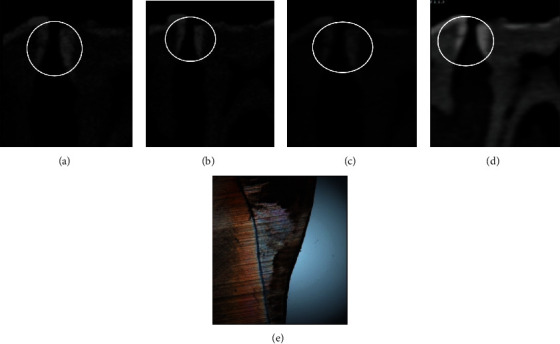
Sagittal CBCT images of the same tooth surface with different exposure parameters. (a) Mode 1: 90 kV_p_, 8 mA, 75 *μ*m. (b) Mode 2: 80 kV_p_, 7 mA, 75 *μ*m. (c) Mode 3: 80 kV_p_, 7 mA, 200 *μ*m. (d) Mode 4: 90 kV_p_, 8 mA, 200 *μ*m. (e) Histological photo of the same surface.

CBCT examination was performed using a Planmeca ProMax 3D Mid device (Planmeca Oy, Helsinki, Finland). Images of each block with and without amalgam restoration were acquired at four imaging modes: imaging Mode 1; 90 kV_p_, 8 mA, 75 *μ*m, imaging Mode 2; 80 kV_p_, 7 mA, 75 *μ*m, imaging Mode 3; 80 kV_p_, 7 mA, 200 *μ*m, and imaging Mode 4; 90 kV_p_, 8 mA, 200 *μ*m (Figures 3(a)–3(d)), both with and without using the Metal Artifact Reduction (MAR) algorithm. The acquired images were taken with a 3.7 cm field of view, voxel size 0.07 mm, and an image acquisition time of 10.8 s. The acquired data were reconstructed into images with a reconstructed sectional interval of 0.2 mm thickness. Observers used the Digital Image Communication in Medicine (DICOM) software to evaluate the reconstructed image slices on the reformatted planes.

### 2.3. Evaluation of Radiographic Images

All radiographs were interpreted by three trained independent oral radiologists with at least 3 years of experience analyzing CBCT images evaluated the images in a room with dim light. All images were analyzed using the OnDemand_3_D™ software (CyberMed, Version 1.0.10.5385, 2015, Seoul, Republic of Korea). All scans were interpreted on an LCD Dell monitor with a 24-inch screen size and 1920 × 1080 high-definition screen resolution. Observers examined all tooth surfaces for the presence of carious lesions on approximal surfaces using a five-point confidence-rating scale: 1 = definitely no lesion, 2 = probably no lesion, 3 = questionable, 4 = probably carious lesion, and 5 = definitely carious lesion.

When carious lesions were deemed present, the observers classified them using a 4-point scale as follows: 0 = sound, 1 = approximal caries in the enamel, 2 = approximal caries extending to the dentino–enamel junction (DEJ) or in the outer half of the dentin, and 3 = approximal caries in the inner half of the dentin [9].

### 2.4. Gold Standard

Prior to observer's interpretation and histological analysis, all 78 tooth surfaces were interpreted via consensus by main authors with different academic levels to confirm all sound and carious surfaces were as preplanned design. Only 5 carious surfaces were excluded.

All 73 included surfaces have undergone histological analysis as the gold standard shown in Figure 3(e).

For histological analysis, tooth crowns were separated longitudinally (mesio–distal direction) at the approximal surfaces. Each tooth surface was examined, and the lesion depths were measured using the following criteria: H0 = sound, H1 = caries less than outer half of enamel, H2 = up to DEJ but not involving it, and H3 = caries in the outer half of dentin.

### 2.5. Statistical Analysis

Statistical analysis was performed using SPSS Version 22. Inter- and intrarater reliability were tested using the interclass correlation coefficient (ICC), with a 95% confidence interval. The Cohen-kappa test was used to assess intra- and interrater agreement. Receiver operating characteristic (ROC) analysis was used to compare the radiographic scores provided by observers with the histology results, and AUC values (Az) were obtained. The areas under the ROC curve were analyzed by pairwise comparison using *z* statistics. Statistical significance was set at a *P* value of ≤0.05.

## 3. Results

Interrater reliability scores, *κ*, are lower for cases with amalgam restorations than without (Table 1).

The ICC showed that the intra-rater reliability ranged between 0.88-0.95 with blocks without amalgam restorations and 0.69-0.90 with amalgam restorations, indicating good to excellent agreement.

Of the 73 surfaces studied, 30 surfaces (41.1%) were sound, 13 surfaces (17.8%) had lesions involving less than half of the enamel thickness, 13 surfaces (17.8%) had lesions involving more than half of the enamel, and 17 surfaces (23.3%) had carious lesions extending into the dentin.

The mean sensitivity, specificity, and area under the curve (AUC) scores for all three readers for approximal caries detection in teeth blocks without amalgam restorations are presented in Table 2. The highest sensitivity, specificity, and AUC were achieved with imaging Mode 1 (90 kV_p_, 8 mA, and 75 *μ*m, i.e., high resolution), while the lowest was achieved with imaging Mode 2 (80 kV_p_, 7 mA, and 75 *μ*m, i.e., high resolution).

Also, Table 2 shows the mean sensitivity, specificity, and AUC scores for all three readers for maximum caries detection in tooth blocks with amalgam restorations, revealing that imaging Mode 4 (90 kV_p_, 8 mA, 200 *μ*m, i.e., low resolution) achieved the highest sensitivity, specificity, and AUC.

Pairwise comparisons between areas under the curve were performed using z statistics. The results showed that there was no statistically significant difference between modes 1, 3, and 4; all showed statistically significantly higher AUCs than Mode 2, with *P* values as follows; Mode 1 vs. 2 < (0.001), 1 vs. 3 (0.651), 1 vs. 4 (0.827), Mode 2 vs. 3 and 2 vs. 4 < (0.001), and Mode 3 vs. 4 (0.753). However, in regard to blocks with and without amalgam restorations, it was found that AUC without amalgam restoration in all modes showed a statistically significantly higher value than with amalgam restorations. The *P* values were as follows: Mode 1 (0.026), Mode 2 < (0.001), Mode 3 (0.016), Mode 4 (0.005).

The fraction of accurate depth predictions in blocks with and without adjacent amalgam restorations are presented in Table 3, which reveals that imaging Mode 3 produced the most consistently good prediction of lesion depth in blocks without amalgam restorations. When amalgam restorations were present, imaging Mode 4 produced the best diagnostic performance.

**Figure 4:**
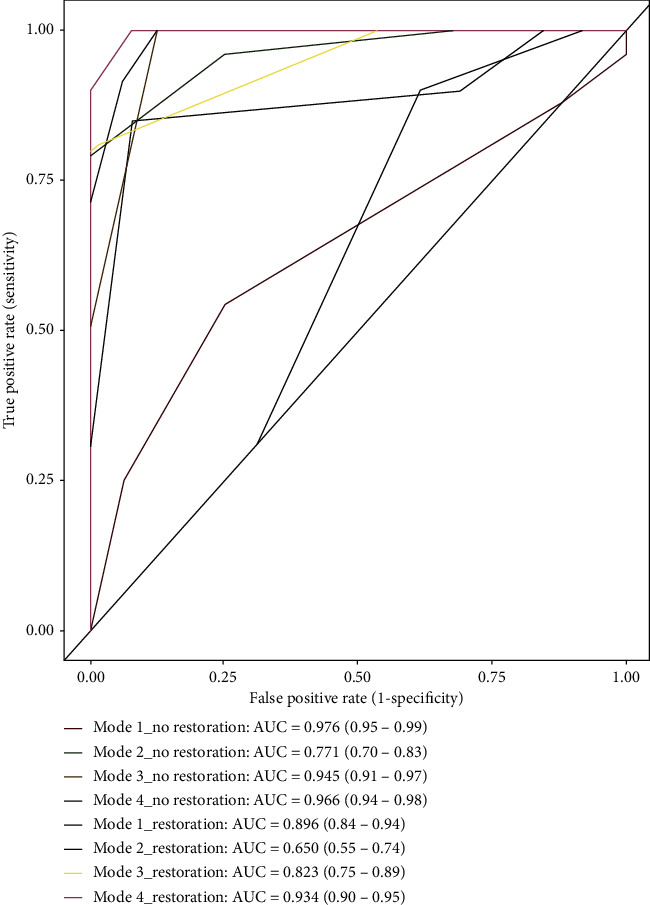
ROC analysis of observers for all imaging modes, with and without amalgam restorations.

ROC analysis was performed to compare the radiographic scores delivered by observers and histological results, and calculated AUC values (*A*_*z*_) are shown in Figure 4. In the evaluation of nonamalgam teeth, the highest *A*_*z*_ value (0.976) was achieved using images produced at imaging Mode 1, 90 kV_p_, 8 mA, and high resolution. While imaging at Mode 2, 80 kV_p_, 7 mA, and high resolution, was inadequate for caries detection in teeth either with or without amalgam restorations.

## 4. Discussion

The objective of this study was to assess the diagnostic accuracy of Planmeca CBCT in detecting noncavitated approximal carious lesions using different imaging parameters and to discern the impact of artifacts produced by amalgam restoration. The working hypothesis of this study was that cross-sectional imaging would provide a higher accuracy than bitewing radiography for the detection of such lesions, as reported by Wenzel et al. [17].

Devices and modalities with sufficient sensitivity and specificity enable caries diagnosis promptly and accurately and avoid unnecessary cavitation surgery in noncaries teeth [5]. In our present study, Planmeca CBCT provided increasingly accurate depth predictions of noncavitated approximal caries as the depth of the lesion increased, especially when the X-ray beam was operated with higher voltage and current. In our case, test lesion depths ranged from 0.76 mm in small enamel lesions to 0.90 mm in dentin lesions. Our overall findings agree with an earlier study [10], found that, in three-dimensional (Accuitomo, NewTom 6-, 9- and 12-inch) fields of view, the ability to detect lesions in the enamel was low (13–21%) and higher for caries in dentin (31–58%).

For all lesions in the present study, we noticed better sensitivity and specificity of the intended CBCT unit, especially at 90 kV_p_, 8 mA, and at low resolution, in cases without beam-hardening artifacts caused by neighboring metallic restorations. This was in harmony with the study by Kamburoğlu et al. [18], who found that the CBCT voxel size did not affect the detection of caries and that CBCT images supported better identification of occlusal and approximal carious lesions than 2D intraoral digital images.

In dental practice, a significant proportion of patients may have metallic tooth restorations, implants, or endodontic restorative materials that can cause beam-hardening artifacts that create bright and dark streaks and noise in the resulting images. Dark bands, in particular, may convey the false impression of a lesion and, therefore, impede caries lesion diagnosis [19, 20].

In our study, none of the interpreted surfaces had amalgam restorations, but some of the neighboring predetermined teeth did. In these latter cases, sensitivity and specificity were improved by using higher exposure parameters and higher resolution. This finding agrees with Haiter-Neto and Wenzel [10], who suggested using small voxels to detect approximal lesions in CBCT sections. Thus, in the present study, we used a 0.07 mm voxel size and a small field of view.

Interobserver agreement for caries detection, calculated using Cohen's *κ* coefficients, was very strong, ranging between 0.84 and 0.93 in teeth without neighboring amalgam restorations, and between 0.77 and 0.91 in teeth with neighboring amalgam restorations. Our finding agrees with Krzyżostaniak et al. [21], who reported interobserver agreement ranging from 0.88 to 0.99 for detection of noncavitated occlusal caries by CBCT.

An in vitro study examines an “idealized” or “much better than clinical” situation and may differ markedly from an in vivo model for CBCT [22]. There are at least two problems: beam-hardening artifacts, and errors due to patient motion, both of which can result in significant image distortion. Additionally, the total X-ray dose that patients receive during a CBCT examination greatly exceeds that received during radiographs with F-speed film. Hence, the CBCT radiographic examinations should be used only when their use can be justified, and radiation exposure should always be minimized [23, 24].

Moreover, although Planmeca CBCT examination can help clinicians to ascertain the true status of approximal noncavitated surfaces of posterior teeth (especially in initial enamel lesions), the routine use of CBCT cannot be recommended in clinical practice and can be used as an auxiliary method for the determination of caries lesions.

## 5. Conclusions

CBCT imaging with higher exposure parameters—90 kV_p_ and 8 mA—is recommended for the detection of initial noncavitated enamel and dentin approximal caries with a statistically significant difference from (80 kV_p_, 7 mA). Increasing the tube voltage (kV_p_) and current (mA) improves diagnostic accuracy in caries detection. The choice of spatial resolution was a more important factor in this regard. Low resolution allowed more accurate detection of approximal caries with adjacent amalgam restorations. While high resolution is recommended in the absence of amalgam restorations, there is a statistically significant difference with and without amalgam with respect to all modes.

## Figures and Tables

**Table 1:** Interrater reliability scores, using Cohen's kappa.

Readers compared	Without amalgam restorations		With amalgam restorations	
	Observed agreement	*κ* (95% CI)	Observed agreement	*κ* (95% CI)
1 and 2	0.953	0.902 (0.848-0.956)^a^	0.910	0.773 (0.670-0.876)^a,b^
1 and 3	0.899	0.791 (0.718-0.865)	0.776	0.467 (0.328-0.605)^b^
2 and 3	0.888	0.768 (0.689-0.848)	0.825	0.600 (0.474-0.726)^b^

^a^The Kappa score for readers 1 and 2 is higher than the other two Kappa scores. ^b^The Kappa score with amalgam restorations is statistically less than the Kappa score for the same readers without amalgam restorations.

**Table 2:** The mean sensitivity, specificity, and AUC scores for all three readers combined for approximal caries detection.

Mode	Sensitivity (true positive rate)		Specificity (1—false positive rate)		AUC (area under ROC curve)		95% CI for AUC	
	Without AR^a^	With AR^b^	Without AR^a^	With AR^b^	Without AR^a^	With AR^b^	Without AR^a^	With AR^b^
Imaging Mode 1 90kV_p_, 8mA, 75*μ*m	0.972	0.966	0.973	0.538	0.976	0.896	0.957-0.994	0.849-0.942
Imaging Mode 2 80kV_p_, 7mA, 75*μ*m	0.875	0.916	0.375	0.256	0.771	0.650	0.704-0.839	0.559-0.742
Imaging Mode 3 80kV_p_, 7mA, 200*μ*m	0.930	0.833	0.812	0.487	0.945	0.823	0.911-0.979	0.755-0.891
Imaging Mode 4 90kV_p_, 8mA, 200*μ*m	0.971	0.983	0.875	0.692	0.966	0.934	0.944-0.986	0.906-0.959

^a^Blocks for teeth without amalgam restorations; ^b^Blocks for teeth with amalgam restorations.

**Table 3:** Fraction of accurate predictions of depth in regard to teeth without and with amalgam restorations.

Mode	True depth = 3				True depth = 2				True depth = 1				No lesion present			
	Without AR^a^ (17 surfaces)		With AR^b^ (17 surfaces)		Without AR^a^ (13 surfaces)		With AR^b^ (13 surfaces)		Without AR^a^ (13 surfaces)		With AR^b^ (13 surfaces)		Without AR^a^ (30 surfaces)		With AR^b^ (4 surfaces)	
	Exact match	2 or 3	Exact match	2 or 3	Exact match	1, 2, or 3	Exact match	1, 2, or 3	Exact match	1 or 2	Exact match	1 or 2	No lesion (0)	0 or 1	No lesion (0)	0 or 1
Mode 1	0.90	0.97	0.86	0.95	0.78	1.00	0.67	0.95	0.76	0.90	0.89	0.94	0.94	0.98	0.69	0.90
Mode 2	0.76	0.88	0.86	0.90	0.33	0.78	0.29	0.90	0.48	0.62	0.61	0.78	0.60	0.88	0.28	0.54
Mode 3	0.76	0.88	0.90	0.95	0.61	1.00	0.52	0.90	0.57	0.81	0.50	0.56	0.90	0.98	0.64	0.90
Mode 4	0.84	0.85	0.86	1.00	0.83	1.00	0.81	0.95	0.76	0.86	0.89	1.00	0.90	0.96	0.85	0.92

^a^Blocks for teeth without amalgam restorations; ^b^Blocks for teeth with amalgam restorations.

## Data Availability

The data used to support the findings of this study are available from the corresponding author upon request.
